# Vulnerability factors of snake bite patients in China

**DOI:** 10.1186/s12889-024-19169-3

**Published:** 2024-06-26

**Authors:** Wenjie Hao, Chuanzhu Lv, Xingyue Song, Lanfen He, Juntao Wang, Yanlan Hu, Yu Chen, Yong Gan, Shijiao Yan, Xiaotong Han

**Affiliations:** 1 School of Public Health, Hainan Medical University, Haikou, Hainan China; 2 Emergency Medicine Center, Sichuan Provincial People’s Hospital, University of Electronic Science and Technology of China, Chengdu, Sichuan China; 3 Research Unit of Island Emergency Medicine, Chinese Academy of Medical Sciences, Hainan Medical University, Haikou, Hainan China; 4 Department of Emergency, Hainan Clinical Research Center for Acute and Critical Diseases, The Second Affiliated Hospital of Hainan Medical University, Haikou, Hainan China; 5 Department of Social Medicine and Health Management, School of Public Health, Tongji Medical College, Huazhong University of Science and Technology, Wuhan, Hubei China; 6 Department of Emergency Medicine, Hunan Provincial Key Laboratory of Emergency and Critical Care Metabolomics, The First Affiliated Hospital, Hunan Provincial Institute of Emergency Medicine, Hunan Provincial People’s Hospital, Hunan Normal University, Changsha, Hunan China

**Keywords:** Snake bite, Vulnerability, China, Antivenom, Access to medical resources

## Abstract

**Objective:**

To analyze the vulnerability factors of snakebite patients in China.

**Methods:**

Multi-stage random sampling was used as the main sampling method and snowball sampling as the auxiliary sampling method. The knowledge, attitude and behavior of snakebite among Chinese residents were investigated. Non-parametric test was used to compare the percentage differences in residents’ knowledge, attitude and behavior of snakebite, and generalized linear regression analysis was used to analyze the influencing factors, and the vulnerability factors of snakebite patients were comprehensively analyzed.

**Results:**

A total of 6338 subjects were included in this study, of which 68.4% were males, and 58.6% were farmers, workers and service personnel. The median total score of knowledge, attitude, and behavior was 26 (22,36). The patients who were improperly treated after injury were ligation proximal to the affected area (23.43%), squeezing (21.82%), and oral and suction wounds (8.74%). Did not go to hospital due to poverty (1351 cases) and did not receive antivenom (2068 cases). There were 21.32% and 32.63%, respectively. Among 4270 patients injected with antivenom 30.7% were vaccinated within 2 h. Among the patients who went to the hospital for treatment (4987), 75.0% arrived at the hospital within 6 h; Among the 4,761 patients who made emergency calls, 37.4% were treated within 0.5 h.

**Conclusions:**

Snakebite patients in China have weak knowledge about snakebite, low awareness of medical treatment, lack of correct prevention and emergency treatment measures, dependence on folk remedies, poor housing and so on. In addition, there are low availability of antivenoms and unreasonable distribution of medical resources in some areas of China. Multisectoral and multidisciplinary cooperation should be developed to prevent and control snakebites in order to reduce the burden caused by snakebites.

**Supplementary Information:**

The online version contains supplementary material available at 10.1186/s12889-024-19169-3.

## Introduction

Snakebites are more common in tropical and subtropical regions, and they particularly affect poor people with the lowest quality of life index [1]. In 2017, the World Health Organization (WHO) classified snakebite as A Category A neglected tropical disease [2]. Around 2.7 million people are bitten by venomous snakes every year [3], causing 8,1410 − 137,880 deaths [4–6], and three times the number of deaths [7, 8]. It is understood that China’s climate is complex and diverse, the area south of the Yangtze River is a high incidence of snake injuries, the annual snake bite incidents are estimated to reach millions, 100,000 to 300,000 people envenomation, the case fatality rate of about 5%, affecting labor producers up to 25–30% [9]. Snakebite is a curable disease, but in countries with inadequate health systems and safe and effective antivenoms, one person dies from a snakebite every five minutes and another four are permanently disabled [6]. The fatality rate from snakebite varies from country to country and is influenced by many factors [10], which may be related to low knowledge and awareness of the population, improper handling, reliance on folk remedies, poor medical practices and lack of timely access to health services [11]. In addition, snakebite patients can be affected by the surrounding population, access to information, the cost of formal treatment, social background, cultural beliefs, and socioeconomic status [12]. In the Chinese study, residents were also found to engage in pre-hospital treatments such as binding, incision detoxification [13], topical application of snake/herbal medicine, superstitious behavior, sucking on wounds, and cupping [14, 15]. Studies have also shown that access to medical resources is difficult for people living in remote areas. For example, in the 1425 cases of venomous snake bites in Nanchang City and surrounding areas, 79.4% of the patients saw a doctor within 12 h [16]. An analysis of snakebite cases in Brazil found that 15.6% of 144,251 snakebite patients failed to receive serum therapy within 6 h of the bite [17]. It is obviously lower than that in China, so it is urgent to understand the current situation of medical resource allocation in China in order to optimize resource allocation and improve the current situation.

WHO has proposed a strategy to halve the burden of venomous bites by 2030 [18]. To achieve this goal, disease prevention and control are the most important steps, and exploring the vulnerability factors of patients and conducting targeted education are key steps. There are studies on snake bite vulnerability abroad [4, 19], but no such studies have been conducted in China. Snakebite vulnerability reflects the influence of individual, social, and programmatic factors associated with snakebite risk, emergency management, and disease outcome [20]. According to the concept of vulnerability constructed by Ayers et al. [21], we divided the vulnerability of snakebite patients into three levels. At the individual level, it refers to the people’s understanding and application of knowledge related to the disease, cultural level and living environment; The social dimension includes infrastructure, transportation network, access to health resources, etc. The program level involves diagnosis and evaluation of diseases. To evaluate the vulnerability factors of snakebite patients in China, and provide the basis for adjusting the allocation of medical resources, formulating health policies and formulating health education programs for residents.

## Materials and methods

### Research object and survey design

We conducted a cross-sectional study in the vicinity of the southern Yangtze River Basin, China, from May 2022 to February 2023 using multistage random and snowball sampling to obtain our study sample. Based on a literature review and our combined work practice, we selected 10 provinces (Fujian, Guangdong, Guizhou, Hainan, Hubei, Hunan, Jiangxi, Sichuan, Yunnan and Zhejiang), one municipality (Chongqing) and one autonomous region (Guangxi) with known incidents of severe snakebites. We used the convenience sampling method to select three cities from each province, municipality and autonomous region: three districts or counties from each city, and three villages from each district or county for a total of 324 residential areas [22]. 

We conducted a survey with between 30 and 50 residents from each community or village using the chance encounter or convenience sampling method. Where literacy levels were low, we provided oral questionnaires. Literate residents self-filled the survey form and returned them to project enumerators. All surveys were distributed and collected face-to-face at the village level. At the same time, we used the instant message software WeChat (Shenzhen Tencent Computer System Co., Ltd, Shenzhen, China) and Tencent QQ (Shenzhen Tencent Computer System Co., Ltd, Shenzhen, China) to show advertisements or pop-up invitations in other regions to recruit random residents to participate in the same online survey we used in the villages.

This study was approved by the medical ethics committee of Hainan Medical University (ethics number: HYLL-2022-226), and informed consent was obtained from each participant before completing the survey or questionnaire. The preface of the survey or questionnaire explained the purpose of the study, and all data were collected without recording any identifying data except for gender, i.e. male or female.

### Questionnaire design and measurement

We survey is based on a combination of domestic and foreign literature research, the consensus of Chinese snakebite experts, interviews with experts and focus group discussions, in order to improve the quality of the questionnaire in three cities in Hainan Province conducted a pre-survey. The questionnaire was designed to assess snakebite knowledge, attitudes, behaviour and health literacy among residents. The questionnaire included: demographic characteristics, knowledge about snakebite, snakebite prevention and first aid behavior, snakebite attitude, snakebite experience, and residents’ health literacy. In this study, residents with snakebite experience were selected and the data of residents’ literacy was removed for analysis.

### Statistical analysis

Excel was used to organize the data, and SPSS22.0 software was used for data analysis. In the knowledge part of the questionnaire, there are 10 questions (multiple choice) with no errors, and each question contains “don’t know”. According to the number of answers selected for each question accounting for 1-25%, 26-50%, 51-75% and 76-100% of the total number of answers (except the “don’t know” option), the score is 1–4 points, and “don’t know” is 0 points. The total score is 40 points. There are 5 questions in the behavior part (multiple choice), some questions have wrong answers, and each question option contains “don’t know”. The score is the same as knowledge. Questions with wrong answers are scored according to the percentage of the number of correct answers to the total number of correct answers. Questions with no correct answers were counted as zero in the total score; and the total score of the behavior part is 16. There are 9 multiple choice questions in the attitude section, all of which contain “don’t know”, and the total score is 9 points. The total modules of knowledge, attitude and behavior are 65 points. Non-normal continuous variables were represented by median (quentile), and categorical variables were represented by frequency and percentage. Non-parametric test was used for comparison between groups. When the variance homogeneity of F test was inconsistent, Welch test was used to test the multicollinearity of independent variables by calculating the variance inflation factor (VIF). A generalized linear regression model was used to investigate the factors affecting residents’ knowledge, attitude and behavior of snakebite. Spearman correlation was used to analyze the correlation among residents’ snakebite knowledge, attitude and behavior scores. When *P* < 0.05, the difference between variables was considered statistically significant.

## Results

### Demographic information of snakebite patients

**Fig. 1:**
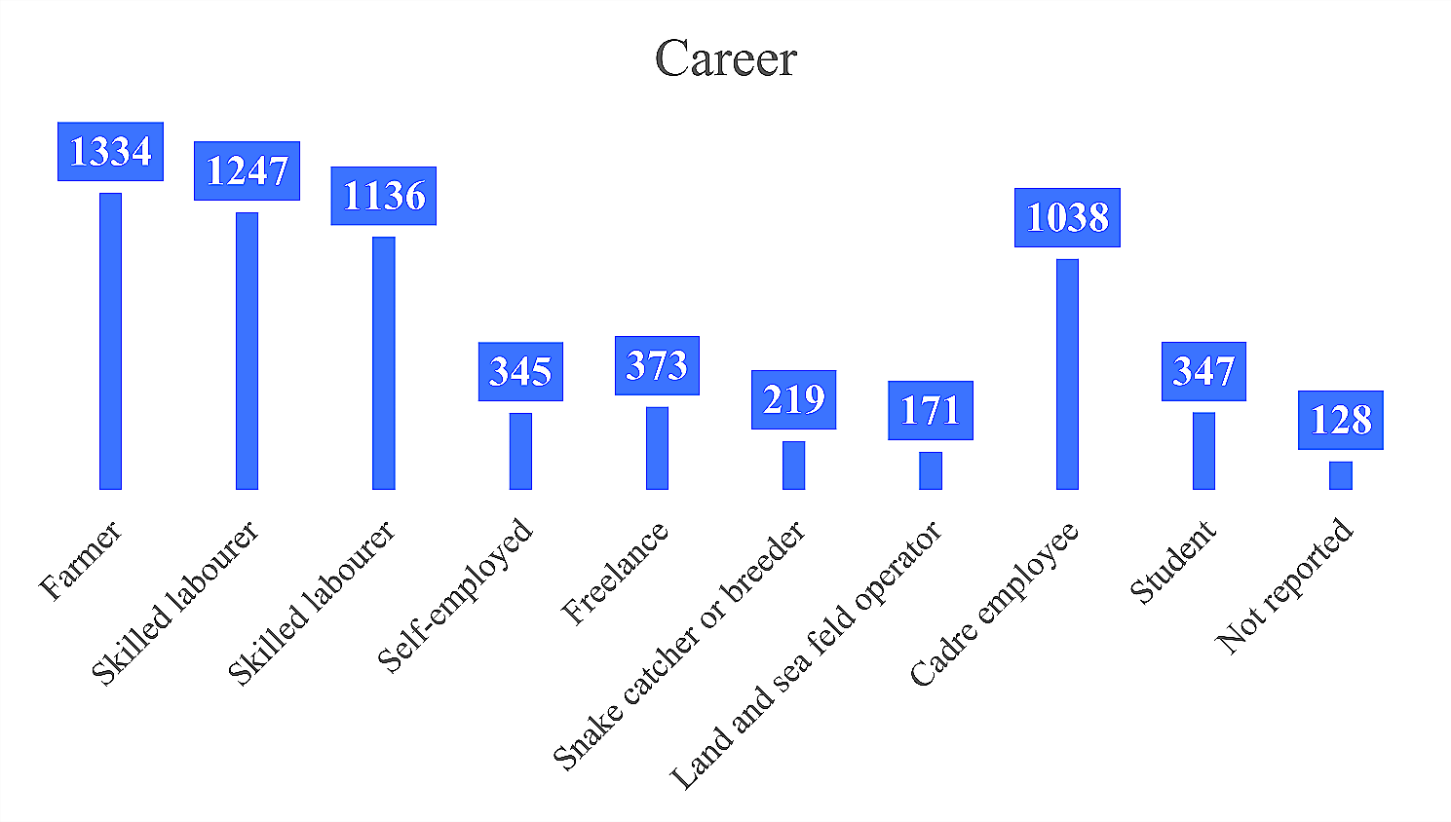
Occupation type of patients

A total of 56804 questionnaires were collected, 1029 were excluded due to logical errors, a total of 6837 residents experienced snakebites, 499 medical personnel were excluded, and finally 6338 questionnaires were included in this study. There were 6338 snakebite patients, of which 68.4% were male and 31.6% were female. The majority were 18–60 years old (89.8%), married and unmarried (90.0%), high school/technical secondary school (66.0%) or less. The majority of snakebite patients were farmers, workers and service personnel (58.6%), as shown in Fig. 1. Social and commercial medical insurance accounted for 37.5%. Among the housing types, wooden houses, tents, bamboo houses and thatched houses accounted for 27.3%. Patients who often went to the field accounted for 97.1%. For details, see Table 1.

**Table 1:** Demographic information of snakebite patients

Characteristic	Number	Percent	Knowledge, attitude, behavior		
			Minimum	Max	M(*P*_25_, *P*_75_)
**All**	6338	100	0	63	26(22,36)
**Gender** ^**a**^					
Male	4333	68.4	0	63	25(22,34)
Female	2005	31.6	0	63	27(22,38)
**Age, year ** ^**a**^					
< 18	421	6.6	0	58	23(20,27)
18~40	4852	76.6	0	63	26(22,35)
41~60	839	13.2	0	58	29(22,40)
> 60	226	3.6	0	60	25(13,41)
**Marital status** ^**a**^					
Married	3294	52.0	0	63	28(23,39)
Single	2403	38.0	0	63	24(21,31)
Divorced	490	7.7	0	55	22(18,28)
Widowed	151	2.3	0	58	23(17,37)
**Education level** ^**a**^					
No education	581	9.2	0	63	23(20,28)
Primary school	899	14.2	0	60	23(20,35)
Middle school	1276	20.1	0	63	25(21,38)
High school or technical secondary school	1428	22.5	0	63	25(22,34)
Vocational institution	1206	19.0	0	63	28(23,37)
Bachelor’s degree or above	948	15.0	0	63	29(23,36)
**Occupation** ^**a**^					
Farmer	1334	21.0	0	63	26(22,38)
Skilled labourer	1247	19.7	0	63	25(22,35)
Service worker	1136	17.9	0	63	26(22,36)
Self-employed	345	5.4	0	58	26(21,35)
Freelance	373	5.9	2	63	27(22,37)
Snake catcher or breeder	219	3.5	0	57	26(22,37)
Land and sea field operator	171	2.7	2	58	29(22,39)
Cadre employee	1038	16.4	0	63	24(22,30)
Student	347	5.5	0	60	26(22,33)
Not reported	128	2.0	5	53	25(21,41)
**Payment method for medical treatment** ^**a**^					
self-pay	1093	17.2	0	63	23(21,31)
Rural cooperative medical care	1992	31.4	0	63	27(22,38)
Social insurance	1510	23.8	0	63	27(22,37)
Commercial insurance	866	13.7	0	61	23(20,30)
Urban medical care	637	10.1	0	63	26(22,36.5)
At public expense	224	3.5	1	58	23(18,35)
Other	16	0.3	28	57	38.5(337.5,42)
**Residence type** ^**a**^					
Cement building	2675	42.2	0	63	26(23,35)
Adobe	1111	17.5	0	63	26(22,38)
Log house	638	10.1	0	61	23(20,27)
Tent	673	10.6	0	54	24(20,33)
Tile-roofed house	790	12.5	0	59	29(22,39)
Bamboo tower	213	3.4	0	57	23(20,28.5)
Thatched cottage	203	3.2	1	49	34(22,40)
Other	35	0.6	30	57	41(36,43)
**Exposure risk** ^**a**^					
Spends majority of time in nature	6155	97.1	0	63	26(22,36)
Little to no time in nature	183	2.9	0	58	21(17,24)

Note: a means *P* < 0.05, the difference is statistically significant

### Knowledge and practice of snakebite

1) The median score of knowledge, letter and action of snakebite patients was 26 (22,36). For details, see Table 1.

2) Among 6338 snakebite patients, 97.0% of them knew at least one preventive measure for snakebite, such as alerting the snake. 13.1% of the patients thought that folk remedies should be used for emergency treatment after injury. 52.1% and 8.7% of the patients knew the combination of traditional Chinese and folk remedy treatment. For details, see Table 2.

**Table 2:** Knowledge and practice regarding snakebite

Characteristic		frequency	%
preventive measure	Cut weeds	2108	42.2
	Realgar fumigation	1836	36.8
	Clothing protection	1817	36.4
	Plant snake repellent plants	1487	29.8
	Repair the rat hole	1217	24.4
	Don’t go out at peak snake times	962	19.2
	Avoid snakes	1069	214
	Escape at once	1130	22.6
	Avoid overgrown areas	885	17.7
	Strike the grass and alarm the snake	865	17.3
	unknown	152	3.0
Distinguish between toxic and toxic	Head shape	2982	47.0
	Snake color	2898	45.7
	Crawling mode	2489	39.2
	Fangs arrangement	1966	31.0
Post-injury management	Minimize activity	2793	44.0
	Catch/kill the snake	2134	33.7
	Ask for help	2482	39.2
	Remember the characteristics of venomous snakes	1932	30.5
	Rinse with soapy water	1804	28.5
	Alcohol, iodophor cleaning	1330	21.0
	Burn 2 to 3 times	856	13.5
	The cupping/syringe draws out the venom	1288	20.3
	Bandage/rope binding	957	15.1
	Branch/splint fixation	960	15.2
	Folk remedy	830	13.1
	Go to the hospita	1010	15.9
	unknown	208	3.3
Treatment method	Western medicine	2421	38.2
	Chinese medicine	2790	44.0
	Integration of traditional Chinese and western medicine	3300	52.1
	Folk prescription	549	8.7
	unknown	265	4.2

### Post-injury results and treatment

**Fig. 2:**
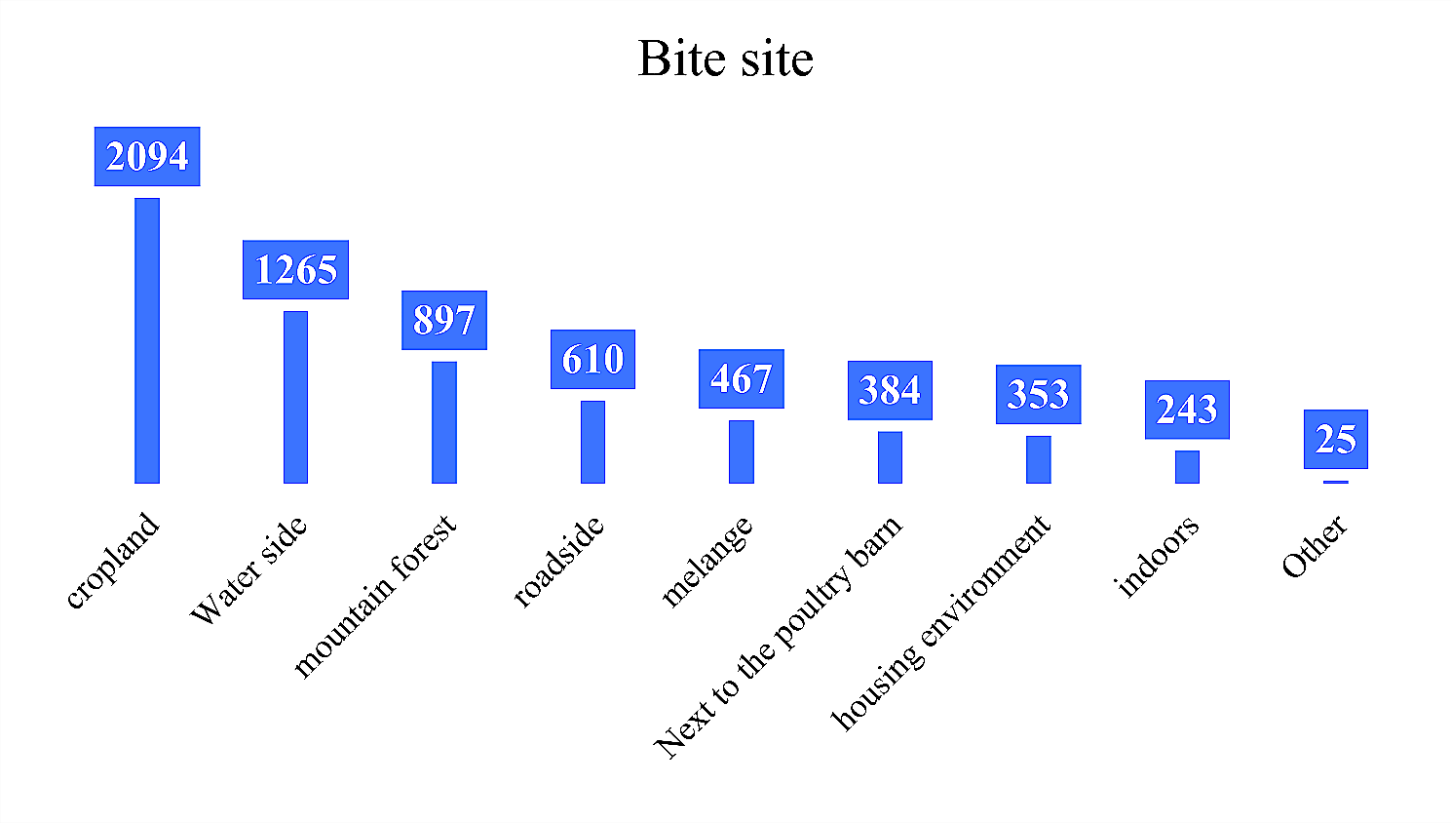
Distribution of bite sites

**Fig. 3:**
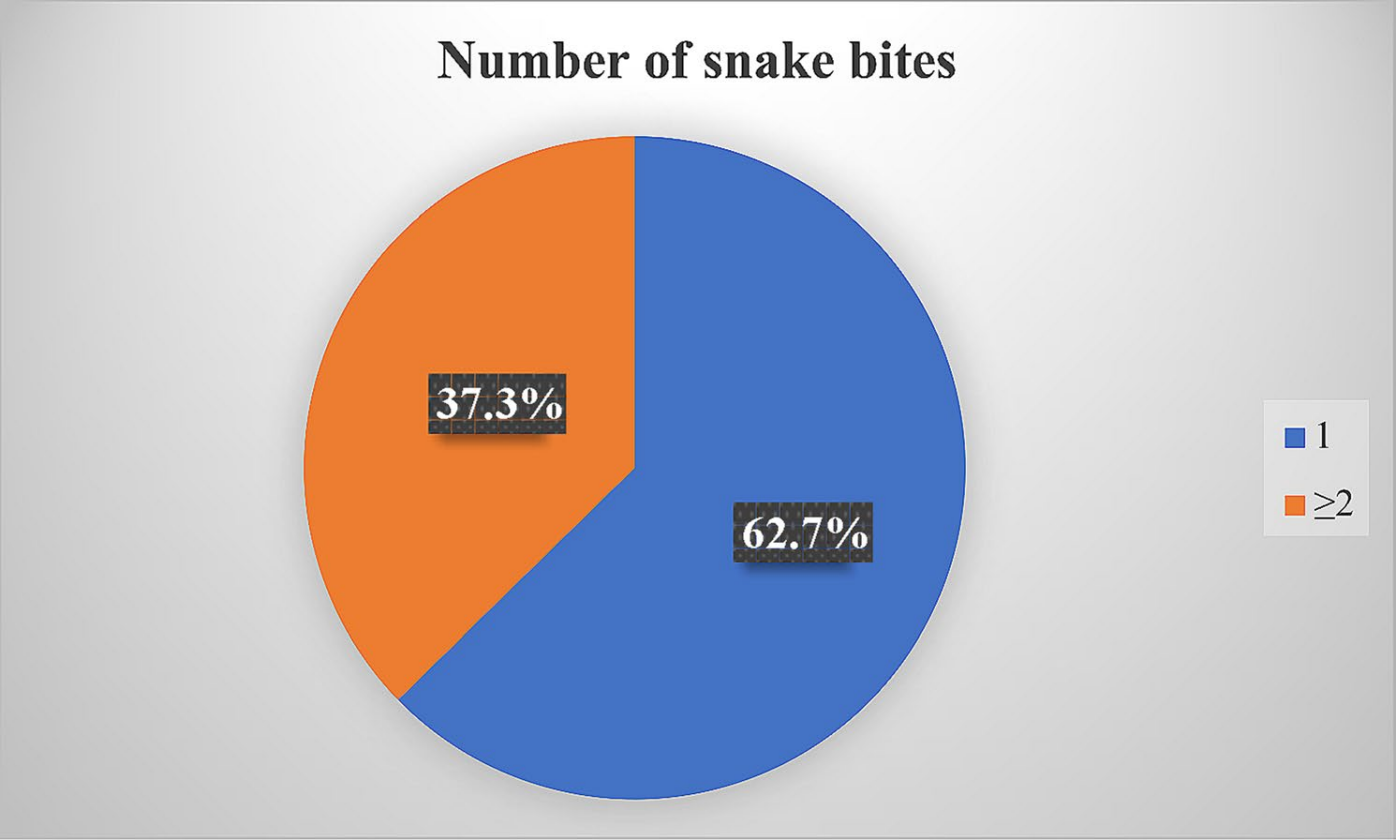
Proportional number of snake bites

(1) Among 6338 snake bite patients, the bite sites were farmland or cultivated land (33.0%) and near water sources (20.0%), as shown in Fig. 2. 81.5% of patients were bitten while farming, walking and outdoor activities. The most common injury sites were hands (18.3%), feet (23.6%), and head and neck (21.3%). 37.3% of the patients had a history of multiple snakebites, as shown in Table 3 and Fig. 3. (2) Improper treatment of 6338 snakebite patients after injury included disinfection with disinfectant (35.26%), ligation of the proximal end of the affected area (23.43%), squeezing (21.82%), and oral suction wounds (8.74%). For details, see Table 4.

**Table 3:** Bite details (*n* = 6338)

Characteristic		Percent(%)
Bite site	cropland	2094(33.0)
	waterside	1265(20.0)
	Mountain forest	897(14.2)
	roadside	610(9.6)
	hag	467(7.4)
	Next to the poultry barn	384(6.1)
	Around the house	353(5.6)
	indoors	243(3.8)
	Other	25(0.4)
Engage in activities	Be a farmer	2347(37.0)
	Walking	1375(21.7)
	Outdoor sports	1446(22.8)
	Sleep indoors	605(9.5)
	Outdoor work	484(7.6)
	Other	81(1.3)
Injured area	Head and neck	1351(21.3)
	Hand	1162(18.3)
	Foot	1496(23.6)
	torso	639(10.1)
	buttock	429(6.8)
	forearm	352(5.6)
	The upper arm	232(3.7)
	calf	510(8.0)
	thigh	167(2.6)
Snake bite history (number of bites)	1	3973(62.7)
	≥ 2	2365(37.3)

**Table 4:** Post-injury treatment

Post-injury management	frequency(%)
No	1850(29.19)
Soapy water rinse	2168(34.21)
Disinfectant disinfection	2235(35.26)
Liquor spraying	1663(26.24)
Proximal ligation	1485(23.43)
extrusion	1383(21.82)
Suction wound	554(8.74)
Suction wound with cupping or syringe	993(15.67)
Incision and bloodletting	885(13.96)
Apply herbs and medicinal wine	849(13.40)
Scorch	760(11.99)
Home disposal	494(7.79)
Remove fingers/toes	221(3.49)

1) Among the patients who went to the hospital for treatment, 75.0% (4987) arrived at the hospital within 6 h, and 30.7% (4270) of the snakebite patients injected with antivenom were vaccinated within 2 h; Among the 4,761 patients who made emergency calls, 37.4% were treated within 0.5 h. For details, see Table 5.

**Table 5:** Availability of health resources

Characteristic	Time(h)	Percent(%)
The timing of the antivenom injection	<2	1312(30.7)
	3–6	1002(23.5)
	6–12	969(22.7)
	12–24	586(13.7)
	>24	340(7.9)
	Not quite clear	61(1.4)
How long to get to the hospital after the injury	<2	1835(36.8)
	2–6	1908(38.2)
	6–12	1017(20.4)
	>12	144(2.9)
	Not quite clear	83(1.7)
The time the ambulance arrived	<0.5	1779(37.4)
	0.5-1	1610(33.8)
	1–2	914(19.2)
	>3	334(7.0)
	Not quite clear	124(2.6)

Note: Injection of antivenom after snake bite (*n* = 4270), trip to hospital (*n* = 4987), arrival time of emergency vehicle (*n* = 4761)

2) 19.25% and 7.94% of snakebite patients who did not go to hospital due to poverty (1351) and did not receive antivenom injection (717), respectively. For details, see Table 6.

**Table 6:** Utilization of medical services after snakebite in Chinese residents: reasons for not receiving serum injection and not going to hospital

Characteristic	Reason	Percent(%)
No antivenoms were used	Cannot afford	57(7.94)
	Be not equipped with	70(9.76)
	Don’t have to	98(13.67)
	I didn’t know there was an antivenom	110(15.34)
	Other	382(53.28)
Did not go to the hospital	Cannot afford	260(19.25)
	Downplay	527(39.00)
	I don’t think I was envenomation	498(36.86)
	Other	66(4.89)

### Generalized linear regression analysis of snakebite patients’ knowledge, attitude and behavior

A multicollinearity test was conducted on the independent variables with statistical significance in the single-factor results of snakebite residents’ knowledge, attitude and behavior scores, and the results showed that (maximum *VIF* = 1.116, minimum *VIF* = 1.007), as shown in the attached table. The generalized linear regression analysis results are shown in Table 7. The scores of snakebite knowledge, attitude and behavior of male residents were lower than those of female residents (*P* < 0.05, *β* =-0.703). Illiterate patients had lower scores than those with a bachelor’s degree or higher education (*P* < 0.05, *β* =-5.100).

**Table 7:** Generalized linear regression analysis of knowledge, attitude and behavior of snakebite patients

Characteristic	β	Standard error	Wald	*p*-value
**Gender (Female)**				
Male	-0.703	0.269	6.838	0.009
**Age, year(>60)**				
< 18	-0.276	0.830	0.110	0.740
18–40	1.577	0.711	4.925	0.026
41–60	2.889	0.755	14.662	0.001
**Marital status** (Widowed)				
Married	2.290	0.836	7.500	0.006
Single	-0.351	0.845	0.172	0.678
Divorced	-1.724	0.924	3.486	0.062
**Education level (Bachelor’s degree or Above)**				
No education	-5.100	0.577	78.041	0.001
Primary school	-2.603	0.483	29.094	0.001
Middle school	-1.575	0.448	12.395	0.001
High school or technical secondary school	-1.187	0.422	7.917	0.005
Vocational institution	0.180	0.430	0.174	0.676
**Occupation (Not reported)**				
Farmer	-0.873	0.924	0.894	0.345
Skilled labourer	-1.838	0.923	3.970	0.046
Service worker	-1.487	0.928	2.568	0.109
Self-employed	-1.784	1.028	3.014	0.083
Freelance	-0.500	1.017	0.242	0.623
Snake catcher or breeder	0.247	1.099	0.051	0.822
Land and sea field operator	1.586	1.155	1.887	0.170
Cadre employee	-2.946	0.931	10.010	0.002
Student	-1.381	1.036	1.779	0.182
**Payment method for medical treatment (**Other)				
self-pay	-10.472	2.513	17.366	0.001
Rural cooperative medical care	-8.695	2.509	12.013	0.001
Social insurance	-8.042	2.509	10.274	0.001
Commercial insurance	-10.881	2.519	18.667	0.001
Urban medical care	-8.882	2.521	12.409	0.001
At public expense	-9.619	2.569	14.014	0.001
**Residence type (**Other)				
Cement building	-13.038	1.712	58.028	0.001
Adobe	-12.985	1.727	56.522	0.001
Log house	-16.584	1.741	90.735	0.001
Tent	-15.061	1.739	74.969	0.001
Tile-roofed house	-12.131	1.734	48.964	0.001
Bamboo tower	-15.144	1.822	69.070	0.001
Thatched cottage	-10.072	1.825	30.467	0.001
**Exposure risk (**Little to no time in Nature)				
Spends majority of time in nature	5.275	0.742	50.533	0.001

Note: *P* < 0.05 was statistically significant

To sum up, the patients were mainly male, farmers, and those who often set foot in the field, and most of them would take improper treatment measures after injury, and the knowledge about snake bite was lacking. The residents have poor access to health resources and weak economic foundation. Education, occupation, and frequent exposure to the wild were factors influencing knowledge about snakebite.

## Discussion

Snakebite mainly affects people living in poor tropical areas [23] with approximately 2.7 million people envenomation by snakebite each year [6, 24]. In order to control the incidence of snakebite, the vulnerability factors of snakebite patients in China were analyzed.

### Individual vulnerability

In this study, the patients were mainly male, farmers, with low education, poor housing [25], and often involved in the field, which was consistent with general epidemiological studies [23]. Among them, males account for 68.4%, which is consistent with Bertolozzi’s study [19, 26]. But different from the high incidence of children and women in Nepal [11], males are generally the main labor force in the family, and have more frequent outdoor work, which has a higher risk. In the multifactorial results, females may have higher scores on snakebite knowledge, belief and behavior than males, whereas there was no statistically significant difference between males and females in the knowledge of symptoms caused by snakebite in the study in Myanmar [27], and there was no significant difference between males and females in their attitudes and reactions to venomous snakes in the study in Indonesia [28]. These differences may be related to regional economic development, cultural beliefs, and sample size, and it is hoped that in the future, the sample size can be expanded and other influencing factors can be fully considered in order to clarify the differences between genders. The research results showed that the majority of patients were farmers, workers and service personnel (58.6%), which was different from that in South Asia (> 50%) [23, 29], which may be due to the significant differences between the natural environment, economic structure and job opportunities of the country or region and rural areas in South Asia. In order to more accurately understand the reasons for this difference, further investigation and research are needed. Compared with highly educated patients, the score of snakebite knowledge with low education may be lower, and lack of awareness is a key factor in the increased mortality [30]. Highly educated patients may have a greater reservoir of knowledge, which makes it easier for them to understand what is known about snakebites. Brazilian scholars have found that a low level of education may be a factor in worsening the outcome of snakebites, as it is associated with a lack of knowledge about prevention and first aid measures [31]. Chinese scholars have found that some patients are reluctant to seek medical treatment after injury due to factors such as low cultural level and language communication barriers [13]. In addition, foreign studies have found that middle-aged men with low education who work outdoors have the highest risk of bites [32]. In a study in India, it was found that more than 85% of residents could master most of the knowledge in the post-training assessment of snake bite knowledge [33], indicating that training activities can help improve residents’ bite prevention and first aid ability.

In summary, patients’ knowledge of snakebite needs to be improved and health education should be continued. However, the high-risk groups are mainly residents of rural areas, who are generally less educated and have a lower ability to accept and absorb new knowledge, so health education activities should be carried out in accordance with the actual situation in different areas. For example, they should be incorporated into school curricula and children should be taught from an early age about the dangers of venomous snake bites and preventive and first-aid measures. In particular, rural schools can improve the awareness of left-behind students about snake bites, affect the awareness of left-behind elderly people and left-behind women, and ensure the life and health of the three types of left-behind people. To prevent and control snakebite incidence, disability and mortality, and to achieve the strategic target of halving the burden of snakebite by 2030.

### Knowledge of snake bites

About 69.6% of residents had no or no correct knowledge of snakebite prevention, a higher proportion than in the Brazilian study [19]. Prevention and appropriate first aid are important public health measures that can reduce the incidence and severity of snakebites, yet studies have found that the majority of the population lacks the proper knowledge of prevention and first aid. In the first aid measures for snake bites, some residents choose to use soap and water to wash the wrong measures, and foreign residents have similar behavior [27]. Chinese scholars have found that washing with soap and water can reduce the damage of mosquito bites [34], which will make residents exaggerate its role, so it is also often used for emergency treatment after bites. However, there is a risk of masking the true condition and delaying treatment. While bandage compression can be applied to neurotic venomous snake bites [35], blind application to venomous snake bites of unknown species may affect prognosis. The general population does not have the ability to identify snake species, so bandages are not recommended [36]. At present, there is no unified view at home and abroad on the on-site first aid of snake wounds. In the 2018 expert consensus in China, it is considered that pressurized and fixed bandaging of bandages can be used as the first aid method for neurotoxic snake bites [37], while the WHO’s Guide to Snake Wounds in Southeast Asia emphasizes that tourniquets, bandages, ropes and other traditional lashing methods should not be used. Tourniquets are still used for first aid treatment by some residents in Nepal (70%) [38] and Sri Lanka (30%) [27]. The risk of complications, disability and death [39] can be increased by the wrong treatment, such as cauterization [40]. 14.0% of patients would choose to apply herbs for wound treatment. Chinese scholars have different views on the impact of applying herbs on the severity of the disease. In this study, 13.4% of the patients would choose to apply herbs or medicinal wine for wound treatment. Some Chinese scholars argue that applying herbs is a favorable factor to reduce the severity [41], while others consider it a risk factor [42]. This may be related to the type of herbs and the use of time, for the pre-hospital application of herbs should be treated dialectically, try not to destroy the wound, so as not to affect the type of snake and the condition of the judgment. In addition, the difference for the results of the study may affect the medical staff’s perception of the treatment methods and influence their diagnosis and treatment. In addition, it can affect the development of health education programs and the effectiveness of health education.

37.3% of snakebite patients had a history of multiple bites, significantly higher than Colombini’s study (6.3%) [31]. A history of multiple bites may be associated with lower risk perception, shallow awareness of protection, disease attitudes, and a lack of local publicity. In addition, 33.7% of patients in the study believed that the causing snake should be caught or killed, and were unfriendly to snakes [43], with the risk of being bitten again. This attitude may stem from misconceptions about snakes, concerns about snake bites, or negative portrayals of snakes in some cultures (snake myths interfere with scientific facts, and film and television portrayals of snakes as cruel animals are misguided) [44]. In a survey in Indonesia, 72% (*n* = 91) of respondents reported that their typical response to an encounter with a venomous snake was to try to kill the snake [28]. It has also been shown that knowledge of snakebite deaths and personal experience of snakebites due to lack of transportation, poor equipment, and distance from medical centers may exacerbate fear of snakes and may increase negative attitudes and destructive behaviors toward snakes [45, 46]. In this case, health education should be relied on to popularize the knowledge of snakes, let people understand the living habits of snakes, the contribution to the environment and how to safely live with snakes, can help reduce people’s fear and misunderstanding of snakes. In addition, inadequate knowledge of snakebite has been shown to be a contributing factor to complications, death, disability or increased treatment costs [33]. In addition, health education by health workers is an effective strategy to reduce the risk of snakebite among residents [10]. Therefore, in order to raise awareness and prevention of snakebite, in response to the strategic plan proposed by the WHO in 2019, disease prevention and control departments, hospitals, public health departments and zoologists should work together to carry out health education with the goal of reducing the risk of snakebite, disability and mortality.

Among the snakebite patients who did not go to the hospital, 39.0% thought it was not serious and did not seek medical treatment, which was also the case abroad [47]. Factors such as personal health awareness, disease judgment, time and severity of symptoms and signs, listening to folk remedies, and knowledge of snakebite may affect the behavior of seeking medical treatment. In addition, because snake bites are common in remote areas with limited access to medical care, local residents tend to trust local doctors more and prefer local methods of treatment. Native doctors often have a high level of prestige and trust in villages. They can act as a bridge between the professional medical team and the villagers in order to facilitate communication, cooperation and joint health education between the two parties to improve the residents’ knowledge of snakebites and their awareness of seeking medical treatment. By making full use of the advantages and resources of both sides, we can provide more comprehensive and effective treatment for snakebite patients, reduce bite risk, disability and mortality, and protect people’s lives.

In summary, residents lack certain knowledge of snakebite prevention, proper first aid treatment, and trust local barefoot doctors. Multi-departmental health education should be contacted to carry out comprehensive health education to improve the relevance and effectiveness of health education. The effect of health education should be evaluated regularly in the later stage to optimize the health education strategy and content, and the influencing factors of health education should be analyzed, and targeted interventions can be carried out on the independent influencing factors in order to strengthen the role of education.

### Access to medical resources

Timely and effective treatment of snakebites is the key to alleviating the physical and mental pain of patients, reducing medical costs, and preventing complications and sequelae. In reality, the high-risk groups of snakebite are those residents of poor areas far from the central city, due to the geographical location and transportation conditions, so that many residents can’t be the first time to the medical institutions after the bite, and can’t obtain timely and effective medical treatment, will further increase the risk of disease deterioration, and increase the vulnerability of patients to disease. In this study, 26.2% of residents who called for emergency ambulance after injury were treated after more than 1 h. WHO has recognized that distance from the source of treatment at a clinic center is a possible barrier to timely treatment, and therefore may be a risk factor for more severe clinical manifestations [48]. In addition, distance leads to delays in treatment, thereby increasing the risk of serious complications, chronic sequelae, and death [49]. In this study, more than 23.3 of patients took more than 6 h to reach the hospital, which is consistent with the Brazilian study [26], and this delay is an independent risk factor for serious complications and related mortality. It may be related to the fact that snakebite victims are mostly residents of remote areas with limited access to resources such as poor transportation facilities and fewer hospitals that carry out snakebite treatment. In a prospective study in Nigeria, it was confirmed that delayed access to health care may lead to poor outcomes [50] 44.3% of patients (those receiving serotherapy) were injected with antivenom after 6 h, consistent with studies of indigenous tribes [49]. WHO recommends that the maximum time for a settlement to reach an antivenom -equipped point is 1 h [51]. Antivenom is currently the only detoxification agent [52], and failure to receive this drug within 6 h will increase the risk of death [53]. In the south of the Yangtze River in China, there may be only one or no hospital in the whole county to reserve antivenoms, and there is a lack of medical resources. In this study, there were patients who did not go to hospital due to poverty (19.25%) and did not inject antivenom (7.94%), which was consistent with foreign studies [54]. Failure to go to the hospital and not injected with antivenoms may be related to the anti-risk ability of families with heavy economic burden, trust local doctors, (37.5% of patients have commercial and social medical insurance), and the price of antivenoms. Due to the relatively high cost of antivenoms and high reserve requirements, poor areas may not be equipped with them [55], which is a big gap with some developed countries [56]. The high cost of key drugs may be related to their difficulty in preparation and lack of effective alternatives. Therefore, governments, businesses and hospitals need to work together. Through optimizing resource allocation, strengthening production and research and development, establishing emergency allocation mechanism, enhancing public awareness, strengthening supervision and quality control and other measures, we can improve the supply capacity and treatment efficiency of antivenoms, and ensure the safety of people’s lives.

Nowadays, our country lacks spatial studies on the high-risk areas of snakebite and the geographical distribution of the most vulnerable people. It is hoped that our scholars can conduct a hotspot analysis of snakebite according to the distribution area of snake species, antivenom availability, distribution of medical centers, and level of economic development. In order to help people identify whether there is a high incidence of snakebite in their area, they can improve their awareness of prevention in order to reduce the risk of snakebite.

## Conclusions

In China, snakebite patients suffer from a lack of relevant knowledge, low awareness of prevention and medical treatment, poor housing and unreasonable distribution of medical resources (antivenoms). Therefore, hospitals, disease prevention and control departments, public health departments and zoology experts should work together to carry out regular health campaigns and bring the campaigns to campuses in order to improve residents’ knowledge of snakebites and their awareness of prevention, as well as to optimize the layout of medical resources and improve the capacity of medical services, so as to reduce the burden of snakebites and control the disability and mortality rates.

### Merits and demerits

This study is the first to explore the vulnerability factors of snakebite patients in China. The survey population is mainly residents of 12 provinces south of the Yangtze River in China, supplemented by residents of other regions. The large survey population makes the results representative. Of course, there are some limitations to this study. Since the survey is based on the history of snakebites of residents over the years, there is recall bias. Second, the collection of information on snakebite patients is not comprehensive, and it is impossible to verify the impact of poor treatment after bite on disease outcome.

### Electronic supplementary material

Below is the link to the electronic supplementary material.

Supplementary Material 1

Supplementary Material 2

Supplementary Material 3

## Data Availability

All data generated or analysed during this study are included in this published article [and its supplementary information files].
