# Supplementary material for: Vulnerability factors of snake bite patients in China
Source: BMC Public Health. 2024 Jun 26;24:1704. doi: 10.1186/s12889-024-19169-3 (PMC11200872; doi:10.1186/s12889-024-19169-3)
Supplement: Supplementary file 1 — Supplementary Material 1 [file 12889_2024_19169_MOESM1_ESM.pdf]

Questionnaire on Knowledge and Behavior of Snakebite in Residents

Greetings.

Thank you very much for taking time out of your busy schedule to fill out this survey. This survey is to understand your knowledge and behavior about snakebites. This survey is anonymous, and the information you provide will be kept strictly confidential, so please rest assured. Please answer according to your actual situation and understanding. If you have any questions about the content of the survey, please consult with the surveyor or healthcare personnel, and please do not miss any questions on the survey.

The last six digits of your ID is_________
Your mobile number is___________

Basic Information

1. Your gender [Single choice]
Male
Female

2. Age [Single choice]
＜18
18-40
41-60
＞60

3. Your marital status [Single choice]
Married
Unmarried
Divorcee
Widowed

4. Your level of education [Single choice]
No education

Primary school
Middle school
High school/Technical secondary school
Junior college
Bachelor degree or above

5. Your current occupation [Single choice]
Farmer
Skilled labourer
Service worker
Self-employed
Freelance
Snake catcher or breeder
Land and sea field operator
Student
Cadre employee
Not reported

6. How you pay for your hospitalization[Single choice]
Self-financed
New Rural Cooperative Medical
Social Insurance
Commercial Insurance
Urban medical care
Public Funds
Other

7. What is your housing type? [Single choice]
Earthen room
Building
Wooden room
Tent
Tile house
Bamboo building
Thatched house
other

8.What are some of the places you regularly dabble in? [Single choice]
Field
Off-field

Snakebite Related Knowledge

1. What you know about preventing snake bites? [multiple choice]
Mowing weeds

Red bezoar
Dress Protection
Plant snake repellent plants
Repairing rat holes
Don't go out during peak snake times
Avoid snake infestations
Escape immediately
Avoid overgrown areas
Strike the grass and alarm the snake
Don't know

2.How can you tell if a snake is poisonous? [multiple choice]
Head shape
Snake body color
Snake crawling style
Snake venom tooth arrangement
Don't know

3.What is the emergency treatment for a snakebite? [multiple choice]
Reduced activity
Catch/kill the offending snake
Call for help
Remember the characteristics of venomous snakes
Soapy water/water rinse
Alcohol, iodophor cleaning
Burn 2~3 times
Suction of venom with cupping/syringe
Bandage/rope ligature
Tree branch/splint fixation
Folk remedy
Go to the hospital
Don't know

4.Do you know what are the treatable forms of snakebite? [multiple choice]
Western Medicine
Chinese Medicine
Combination of Chinese and Western Medicine
Folk remedy
Don't know

5. Do you know about antivenom? [Single choice]
Yes
No

6. Do you know which provinces have the highest incidence of snakebites? [multiple choice]
Guangdong province
Guangxi province
Yunnan province
Hubei province
Fujian province
Hunan province
Jiangxi province
Guizhou province
Don’t know

7. What kinds of snakes do you know or understand? [multiple choice]
Venomous snake
Forest or meadow viper
Trimeresurus stejnegeri
Cobras
Golden annular snake
Deinagkistrodon
Sea serpent
Silver ring snake
Don’t know

8. What do you think is the season for snake activity? [multiple choice]
Spring (March-May)
Summer (June-August)
Fall (September-November)
Winter (December-February)
Don’t know

9. What do you think of the specific time period of the snake event? [multiple choice]
Daytime
Early morning
Dusk
Night
Don't know

10. Do you know the habits of snakes? [multiple choice]
Lives in bushes or bamboo forests in hilly and mountainous areas, hillside burial mounds, etc
Near fish ponds, rice paddies, roads and homes
Mainly out and about during the day
Wide-ranging diet, can eat fish, frogs, lizards, snakes, birds, bird eggs, rodents and so

on
Snakes have a habit of hibernating
Don't know

11. What do you think may be the body's uncomfortable reaction to a snake bite? [multiple choice]
There are 2 teeth marks in the wound
Redness, swelling, bleeding
Pimple
Dizziness, headaches
Nausea, vomiting
Unconscious
Respiratory distress
Blurred vision
Fever, chest tightness, shortness of breath
Nosebleeds, blood in urine, blood in stool
Don't know

12. What complications do you think are associated with venomous snake bites? [multiple choice]
Wound infection
Tissue necrosis
Wound ulcers
Physical dysfunction
Toxic shock
Kidney failure
Cardiac arrest
Don't know

13. Do you consider snakebite to be a serious disease? [Single choice]
Yes
No
Don't know

14. Did you know that snakebites can be disabling?
Know
Don’t know

15. Did you know that snakebites can be fatal? [Single choice]
Know
Don’t know

16.Did you know that snakebites can cause psychological disorders? [Single choice]

Know
Don’t know

17. Do you think there is a need to popularize the knowledge related to self-rescue of poisonous snake bites? [Single choice]
It's very necessary
It's necessary
Ordinary
It's not necessary

18. What form of snakebite education would you like to receive? [multiple choice]
Small video
TV
Offline lectures
Books or related manuals

19. Would you like to be trained in snakebite self-rescue knowledge and skills? [Single choice]
Willing
Not willing
Don't know

20. What do you think is the first thing to do after a snake bite？ [multiple choice]
Get out of the snakebite environment immediately and do not attempt to catch or chase the snake to avoid secondary bites
Try to memorize the characteristics of the snake or take pictures
Stay calm, avoid panic, and minimize movement of the injured extremity
Remove all kinds of restricted objects from the injured area so that they cannot be removed due to subsequent swelling, aggravating local damage
Get out of here!
Bandage pressure immobilization can be used for neurotoxic venomous snake bites to avoid too tight and prolonged compression leading to necrosis of the limb due to ischemia live
Flush the wound with clean water and call an ambulance for early evacuation to a hospital that is able to treat the wound.
Don’t know

21. Do you know how to flush out a snake bite? [multiple choice]
Flush the wound with soapy water or alcohol for at least 15 minutes
Rinse with vinegar or soy sauce for 15 minutes
Toothpaste on the wound

Soak the wound in soapy water for at least 15 minutes
Don’t know

22. When a venomous snake bite occurs, which of the following symptomatic resuscitation measures do you think is correct? [multiple choice]
For patients with respiratory distress, give artificial respiration
Patients with persistent bleeding wounds, give appropriate dressings to reduce bleeding
For unconscious patients, treat them as comatose and observe the patient's respiration
Perform cardiopulmonary resuscitation on a nonbreathing patient
Immediately draw out the toxin by fire cupping after a snake bite
Don’t know

Snakebite experience

1. Have you ever been bitten by a snake? [Single choice]
Yes
No

2.What activity you were engaged in when you were bitten? [Single choice]
Farming
Taking a walk
Outdoor sports
Sleeping indoors
Outdoor work
Other

3. Where did you bite? [Single choice]
Head and neck
Hands
Feet
Trunk
Buttocks
Forearm
Upper arm
Calf
Thighs

4.Where was the bite? [Single choice]
Farmland or arable land
Near a water source
Mountain forest
Roadside

Debris pile
Next to a poultry enclosure
Around the house
Inside the house
Other

5.What is your pain level after a snakebite? [Single choice]
0 points No obvious pain
Mild pain on a scale of 1-3 (does not interfere with sleep)
4-6 pain (interferes with normal sleep)
7-10 points of pain (severe sleep disruption)

6. How many times have you been bitten by a snake (including this time)? [Single choice]
1
≥2

7.Do you choose to attack the snake or stay away from it in the event of a snakebite? [Single choice]
Tackle
keep away from

8.What is the emergency treatment you take after being bitten by a snake? [multiple choice]
None
Rinsing with clean water
Disinfectant solution
White wine spraying
Proximal terminal ligation
Squeezing
Oral suction of wound
Fire can or syringe to suck the wound
Incision and bleeding
Application of herbs and medicinal wine
Cauterizing
Home treatment
Removal of fingers/toes

9.Did you call an ambulance after being bitten by a snake? [Single choice]
Yes
No

9.1 How long did it take to get treatment after calling an ambulance? [Single choice]

＜0.5h
0.5-1h
1-2h
＞3h
Unknown

10.Did you visit a medical facility after being bitten by a snake? [Single choice]
Yes
No

10.1 why you did not seek medical attention after being bitten by a snake? [Single choice]
Unaffordable
Considered not serious
Considered not envenomated
Other

10.2 How long does it take to reach a medical facility after a snakebite? [Single choice]
＜2h
2-6h
6-12h
＞12h
Unknown

11. Have you been vaccinated with antivenom after being bitten by a snake? [Single choice]
Yes
No

11.1 Why don't you inject antivenom? Single choice]
Unaffordable
Not equipped with antivenom
Not necessary
Did not know there was an antivenom

11.2 How soon after the bite do you get antivenom? [Single choice]
＜2h
3-6h
6-12h
12-24h
＞24h
Unknown

12. Whether there are residual sequelae (diagnosed by the hospital)？ [Single choice]

Yes

No
