# Supplementary material for: Vulnerability factors of snake bite patients in China
Source: BMC Public Health. 2024 Jun 26;24:1704. doi: 10.1186/s12889-024-19169-3 (PMC11200872; doi:10.1186/s12889-024-19169-3)
Supplement: Supplementary file 2 — Supplementary Material 2 [file 12889_2024_19169_MOESM2_ESM.docx]

**Schedules**

**Multicollinearity results**

| **Variant** | ***β*** | **Standard error** | **Tolerance** | ***VIF*** |
| --- | --- | --- | --- | --- |
| Gender | 1.171 | 0.276 | 0.988 | 1.012 |
| Age | 1.453 | 0.227 | 0.976 | 1.025 |
| Marital status | -2.182 | 0.181 | 0.923 | 1.084 |
| Education level | 0.937 | 0.089 | 0.896 | 1.116 |
| Occupation | 0.126 | 0.063 | 0.9 | 1.111 |
| Payment method for medical treatment | -0.071 | 0.098 | 0.912 | 1.097 |
| Residence type | 0.231 | 0.073 | 0.933 | 1.072 |
| Exposure risk | -5.953 | 0.764 | 0.993 | 1.007 |
